# Cingulate island sign temporally changes in dementia with Lewy bodies

**DOI:** 10.1038/s41598-017-15263-2

**Published:** 2017-11-07

**Authors:** Tomomichi Iizuka, Rui Iizuka, Masashi Kameyama

**Affiliations:** 1 Center for Dementia, Fukujuji Hospital, Japan Anti-Tuberculosis Association, 24-1-3, Matsuyama, Kiyose-City, Tokyo, 204-8522 Japan; 2 Department of Biology, Waseda University, 1-104 Totsukamachi, Shinjuku-ku, Tokyo, 169-8050 Japan; 3 Division of Nuclear Medicine, Department of Radiology, School of Medicine, Keio University, 35 Shinanomachi, Shinjuku-ku, Tokyo, 160-8582 Japan; 4 Department of Radiology, Tokyo Metropolitan Geriatric Hospital, 35-2 Sakaecho, Itabashi-ku, Tokyo, 173-0015 Japan

## Abstract

The cingulate island sign (CIS) that reflects sparing of the posterior cingulate cortex (PCC) relative to the precuneus plus cuneus on FDG-PET and brain perfusion SPECT, has been proposed as a feature of dementia with Lewy bodies (DLB). As the CIS is influenced by concomitant Alzheimer’s disease (AD)-type neurofibrillary tangle (NFT) pathology, we postulated that the CIS gradually disappears as DLB progresses. To determine temporal changes in the CIS, 24 patients with mild DLB and 7 with prodromal DLB underwent ^123^I-IMP–SPECT and MMSE twice at an interval of two years. The CIS was evaluated as a ratio that was derived by dividing IMP accumulation in the PCC with that in the precuneus plus cuneus. We found that the CIS changed over time and that the relationship between CIS ratios and MMSE scores was inverted U-shaped. Thus, the CIS was most obvious in the vicinity of an MMSE score of 22 and it gradually diminished as the MMSE score decreased. Moreover, a lower CIS ratio in mild DLB was associated with a worse prognosis for cognitive decline, presumably due to concomitant AD-type NFT pathology. Our findings would provide a foundation for the appropriate usage of CIS as a biomarker.

## Introduction

Dementia with Lewy bodies (DLB) shows various clinical manifestations. The revised criteria^1^ include the core-features of fluctuating cognitive function, recurrent visual hallucinations, spontaneous parkinsonism and rapid eye movement sleep behavior disorder. Variations in the clinical picture of DLB can be attributed to the various degrees of Lewy body pathology and coexisting Alzheimer disease (AD) pathology that are found at autopsy^2–4^.

Some neuroimaging features of DLB have been proposed to enhance diagnostic accuracy. Occipital hypometabolism on ^18^F-FDG-PET images is a useful biomarker that can discriminate DLB from AD^5–8^ and it is listed as a supportive biomarker in the revised criteria^1^. Hypometabolism in the occipital cortex is accompanied by cholinergic dysfunction^9,10^ and white matter disruption^11^ that have been attributed to underlying cortical-subcortical Lewy body pathology^11–15^.

The cingulate island sign (CIS) on FDG-PET and brain perfusion SPECT has recently been proposed as a neuroimaging feature of DLB^16,17^. The term refers to sparing of the posterior cingulate cortex (PCC) relative to the precuneus plus cuneus (PpC). This sign is highly specific for an accurate diagnosis of DLB^16,17^ and was described as a supportive biomarker in the revised criteria^1^. However, precise behavior of CIS, which is needed for CIS-based diagnosis, has not been fully understood.

Previous linear analysis did not uncover the association between the CIS and general cognition assessed by Mini-Mental State Examination (MMSE)^18^ and Mattis Dementia Rating Scale^19^. As the CIS have been reported mostly in patients with mild DLB^16–19^, its presence in patients with more advanced DLB and whether it temporally changes remain unknown.

The CIS is reportedly influenced by AD-type neurofibrillary tangle (NFT) pathology^19^ and we reinforced the notion by showing relationship between CIS and medial temporal lobe (MTL) atrophy^18^. We therefore postulated that CIS would disappear as DLB progresses, because the AD-type NFT pathology increases over time^20–22^. To examine the hypothesis, we prospectively followed-up CIS in patients with prodromal and mild DLB for two years using brain perfusion SPECT.

## Results

### The CIS temporally changed

**Figure 1:**
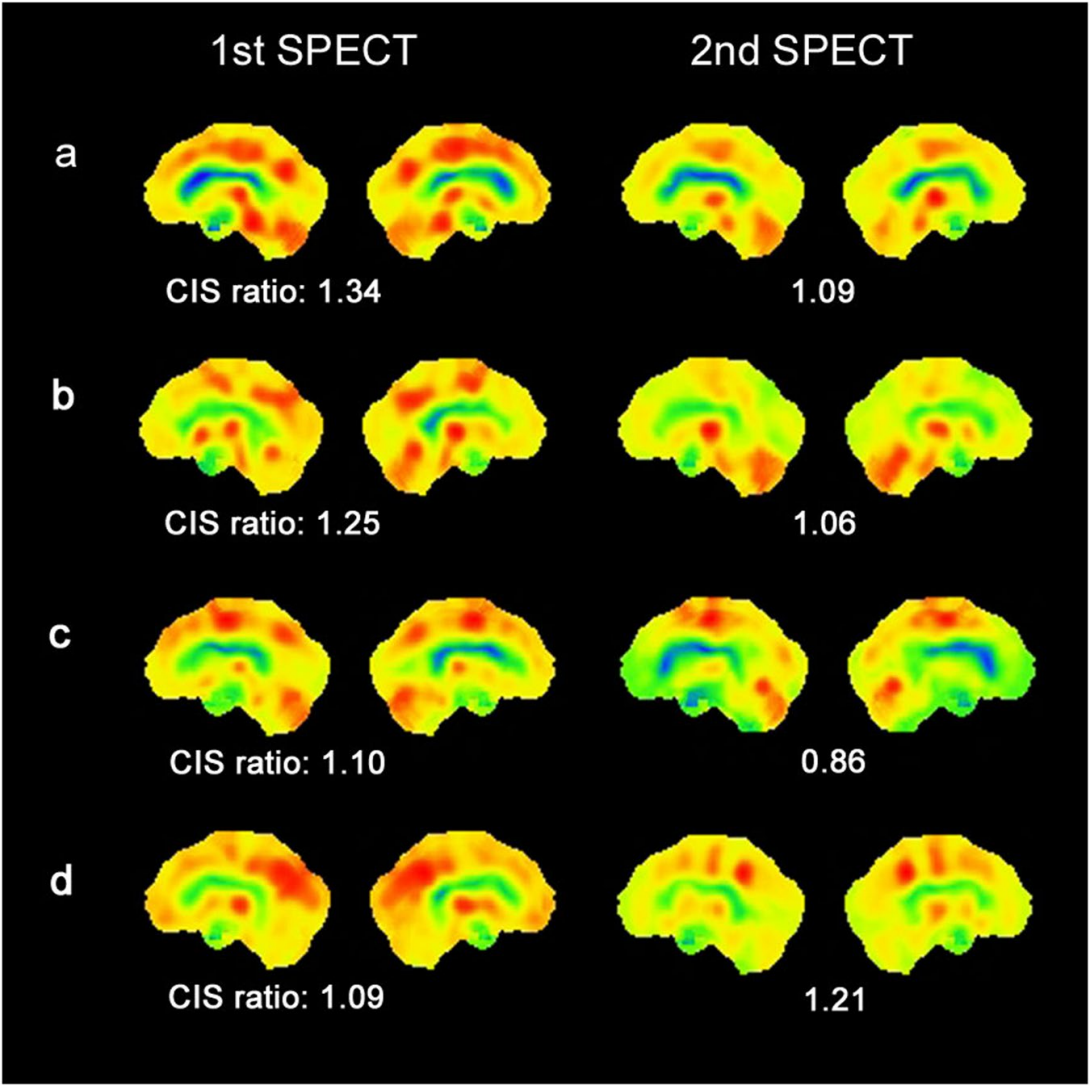
Temporal changes in CIS ratios in 3D brain perfusion images from 4 patients. Values below each brain image are CIS ratios. (**a**) Male patient with mild DLB aged 75 years with higher initial CIS ratio. The 2nd SPECT evaluation shows that CIS has become unclear, but CIS ratio remains over 1.00. MMSE scores at 1st and 2nd SPECT were 22 and 19, respectively. (**b**) Male patient with mild DLB aged 74 years with higher initial CIS ratio that became unclear two years later, although it remained over 1.00. MMSE scores at 1st and 2nd SPECT were 22 and 20, respectively. (**c**) Male patient with mild DLB aged 78 years with lower 1st CIS ratio. MMSE scores at 1st and 2nd SPECT were 23 and 18, respectively. (**d**) Female patient aged 76 years with prodromal DLB. At 1st SPECT, CIS was unclear but became obvious at 2nd SPECT. MMSE scores at 1st and 2nd SPECT were 25 and 21, respectively.

**Figure 2:**
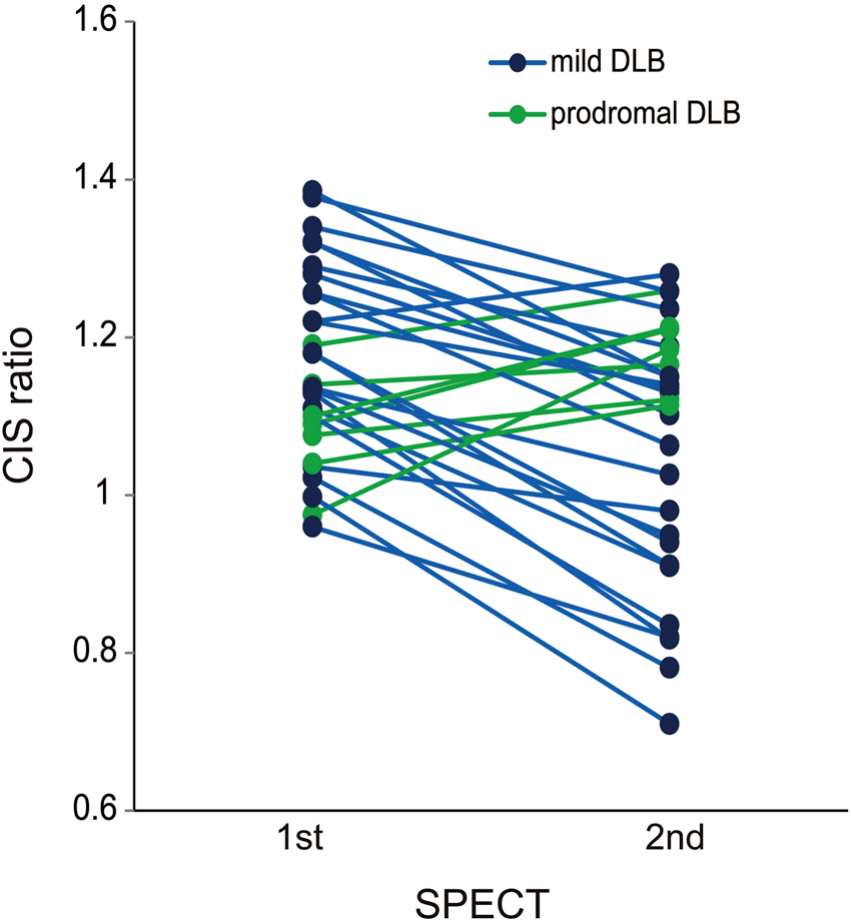
Changes in CIS ratio between 1st and 2nd SPECT assessment. The CIS ratios significantly decreased in patients with mild DLB during two years of follow-up (*blue*; paired *t*-test: *t* = 6.525, df = 23, *p* < 0.001), but significantly increased among seven patients with prodromal DLB (*green*; paired *t*-test: *t* = −3.625, df = 6, *p* = 0.011).

To determine temporal changes in the CIS, 24 patients with mild DLB and 7 others with prodromal DLB underwent twice of N-isopropyl-p-[^123^I] iodoamphetamine (^123^I-IMP) brain perfusion SPECT at an interval of two years. The 22 persons with normal cognition were assessed with^123^I-IMP-SPECT once. The CIS was evaluated by CIS ratio that was derived by the division of the IMP accumulation in the PCC with that in the PpC. Figure 1 shows temporal changes of CIS in 4 patients with DLB. Prodromal and mild DLB progressed to mild DLB and mild to moderate DLB during the two-year follow-up, respectively. The MMSE scores were significantly lower in patients with DLB than with persons with normal cognition (Table 1) and decreased in all patients with DLB during the two-year follow-up. The CIS ratios of patients with mild DLB were significantly decreased at the time of the 2nd assessment (Fig. 2; paired *t*-test: *t* = 6.525, df = 23, *p* = 0.001). The CIS ratio significantly increased in 7 patients with prodromal DLB (paired *t*-test: *t* = −3.625, df = 6, *p* = 0.011). All DLB patients except 2 prodromal DLB patients showed abnormal striatal dopamine transporter (DAT) density at 1st assessment with DAT-SPECT. The DAT density in the 2 patients became abnormal at the 2nd DAT-SPECT after two years.

**Table 1:** Demographic features of patients with DLB.

1st CIS ratio	DLB				normal
	total	prodromal	mild		
			higher	lower	
Number (n)	31	7	12	12	22
Male/Female	17/14	5/2	6/6	6/6	12/10
Age (years)	73.9 ± 3.7	74.4 ± 3.3	73.7 ± 3.8	73.7 ± 4.2	74.1 ± 1.6
Educational (years)	14.3 ± 2.2	15.1 ± 2.3	14.7 ± 2.1	13.8 ± 2.3	13.9 ± 2.4
Disease duration (months)	8.0 ± 4.8	3.4 ± 1.9^†^	9.1 ± 4.3	9.6 ± 5.0	NA
1st MMSE score	22.9 ± 1.9^*^	25.3 ± 0.5^†^	22.7 ± 1.0	21.9 ± 1.9	29.4 ± 0.5
2nd MMSE score	18.6 ± 2.5^*^	20.6 ± 1.5^†^	19.5 ± 1.7^†^	16.7 ± 2.0	NA
DAT binding	2.36 ± 1.03	2.82 ± 1.32	2.10 ± 0.67	2.34 ± 1.08	NA
1st CIS ratio	1.17 ± 0.14^*^	1.09 ± 0.07	1.29 ± 0.06^†^	1.11 ± 0.09	1.08 ± 0.25
2nd CIS ratio	1.06 ± 0.17	1.18 ± 0.05^†^	1.17 ± 0.07^†^	0.90 ± 0.10	NA
1st MTL atrophy	1.37 ± 0.55^*^	0.83 ± 0.22^†^	1.16 ± 0.32^†^	1.89 ± 0.40	0.51 ± 0.33
2nd MTL atrophy	1.55 ± 0.68^*^	0.89 ± 0.24^†^	1.25 ± 0.36^†^	2.24 ± 0.46	NA

Mean ± SD; NA, not assessed; Disease duration, duration between onset of symptoms and 1st assessment; MTL atrophy, evaluated by VSRAD score; ^*^ *p* < 0.05, significant difference compared with normal group by two sample *t*-test; ^†^ *p* < 0.05, significant difference compared with mild DLB with lower initial CIS ratio by Tukey-Kramer multiple comparison test.

### Prognosis for cognitive decline in mild DLB with a higher and a lower initial CIS ratio

**Figure 3:**
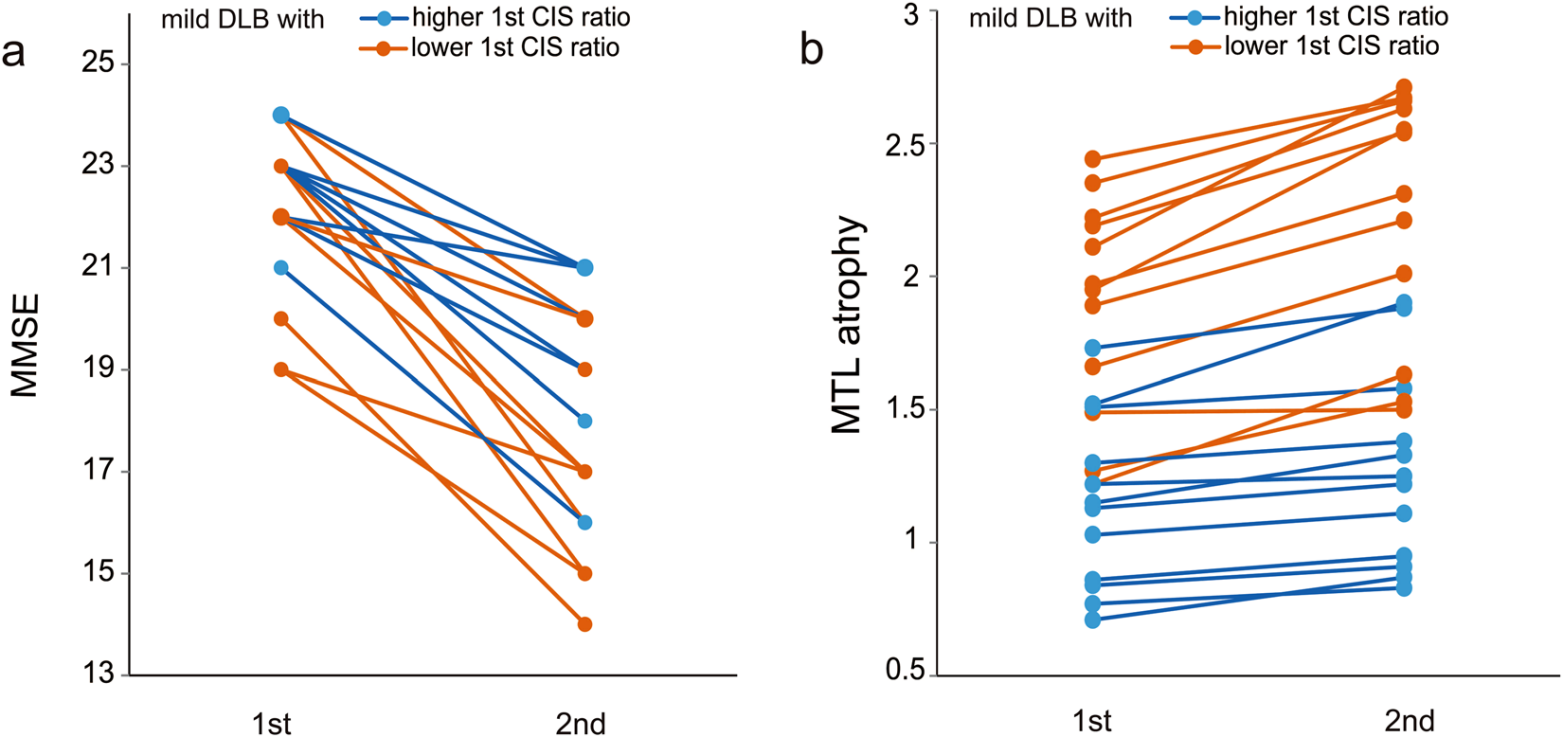
Changes in MMSE scores and MTL atrophy in mild DLB. (**a**) Changes in MMSE scores. During two years of follow-up, MMSE scores decreased more in group with lower, than higher initial CIS ratio. The repeated measures ANCOVA with age and years of education as potential confounding covariates confirmed a significant group effect [*F* (1, 20) = 5.601, *p* = 0.028]. (**b**) Changes in MTL atrophy. The repeated measures ANCOVA with age and years of education as potential confounding covariates revealed the group effect on change in MTL atrophy [*F* (1, 20) = 14.439, *p* = 0.001]. Thus, MTL atrophy in the group with lower CIS ratio progressed more than that with higher CIS ratio during two-year follow-up.

We assigned the patients with mild DLB into groups according to whether they had higher or lower CIS ratios at the initial SPECT assessment. We found a more obvious temporal decrease in the MMSE in the group with a lower, than a higher initial CIS ratio (Fig. 3a). MMSE scores in both groups significantly decreased by paired *t*-test (group with higher initial CIS ratio: *t* = 6.44, df = 11, *p* < 0.001; group with lower initial CIS ratio: *t* = 7.16, df = 11, *p* < 0.001). The repeated measures analysis of covariance (ANCOVA) with age and years of education as potential confounding covariates confirmed a significant group effect [*F* (1, 20) = 5.601, *p* = 0.028].

**Figure 4:**
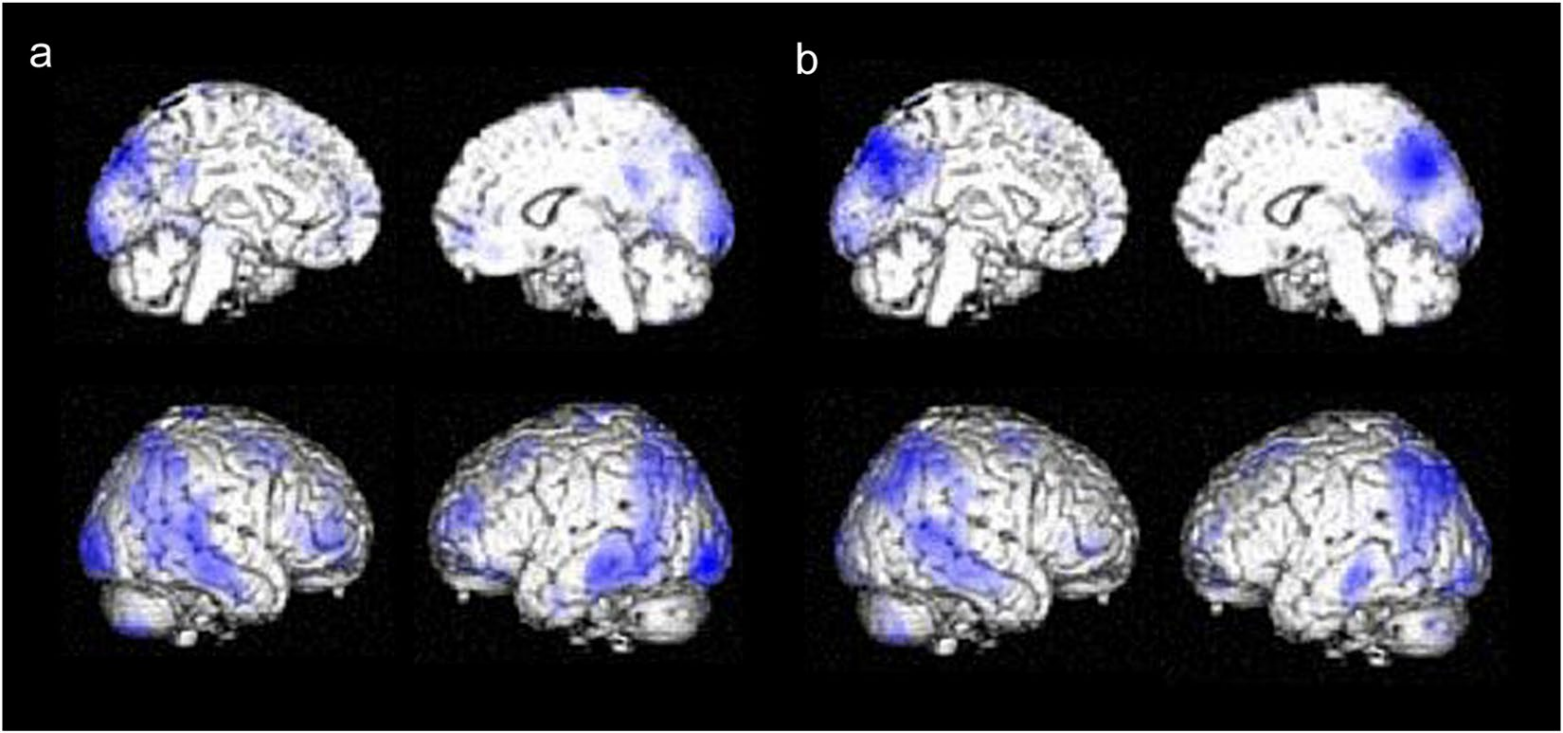
Decrease in rCBF during two years in mild DLB with higher and lower initial CIS ratios. Brain regions in which rCBF decreased during two years in mild DLB patients were analyzed with SPM12 (**a**) mild DLB patients with higher initial CIS ratios; (**b**) those with lower initial CIS ratios (*p* = 0.001, uncorrected). The rCBF decrease in PCC was more evident in mild DLB patients with lower initial CIS ratios than in those with higher CIS ratios. It was proved by ROI analysis with repeated measures ANCOVA with age and years of education as potential confounding covariates [*F* (1, 20) = 8.068, *p* = 0.01].

Decrease in regional cerebral blood flow (rCBF) during two years in mild DLB patients with higher and lower initial CIS ratios was analysed with SPM12 and is demonstrated in Fig. 4. The rCBF decrease in PCC was more evident in mild DLB patients with lower initial CIS ratios than in those with higher CIS ratios. It was supported by the finding that the rCBF ratio in the PCC ROI was more significantly reduced in mild DLB patients with lower initial CIS ratios than in higher initial CIS ratios by repeated measures ANCOVA with age and years of education as potential confounding covariates [*F* (1, 20) = 8.068, *p* = 0.01].

Having a higher and a lower initial CIS ratio in mild DLB was associated with 3.1 ± 1.3 and 5.0 ± 1.9 points reduction in MMSE scores during two-year follow-up, respectively. We have selected initial MMSE score, initial MTL atrophy score and years of education as variates for multiple regression analysis to predict CIS change. It revealed that initial MMSE score and initial MTL atrophy score significantly influenced temporal changes in the CIS ratio (Table 2).

**Table 2:** Associations with the change in CIS ratio by multiple regression analysis.

	B	SE	*β*	*t*	*p*
1st MMSE	0.028	0.012	0.386	2.403	0.023
1st MTL atrophy	−0.080	0.033	−0.254	−2.392	0.024
Education	0.013	0.008	0.254	1.534	0.137

Target variable: change in CIS ratio**;** *R* ^2^ = 0.688, adjusted *R* ^2^ = 0.653, *F*(3, 27) = 19.825, *p* < 0.001; All of the age, gender, years of education, 1st MMSE score, 1st MTL atrophy score and DAT density were examined by forward-backward selection and above three variables were selected as a model with maximum adjusted *R* ^2^; B, partial regression coefficient; SE, standard error; *β*, standardized partial regression coefficient.

During two-year follow-up, Voxel-Based Specific Regional Analysis System for Alzheimer’s Disease (VSRAD)-scores for MTL atrophy significantly increased in both groups by paired *t*-test (group with higher initial CIS ratio: *t* = −2.50, df = 11, *p* = 0.029; group with lower initial CIS ratio: *t* = −7.06, df = 11, *p* < 0.001; Fig. 3b). The repeated measures ANCOVA with age and years of education as potential confounding covariates revealed the group effect on change in MTL atrophy [*F* (1, 20) = 14.439, *p* = 0.001]. Thus, MTL atrophy in the group with lower CIS ratio progressed more than that with higher CIS ratio during two-year follow-up.

### The relationship between CIS ratios and MMSE scores was an inverted U-shape

**Figure 5:**
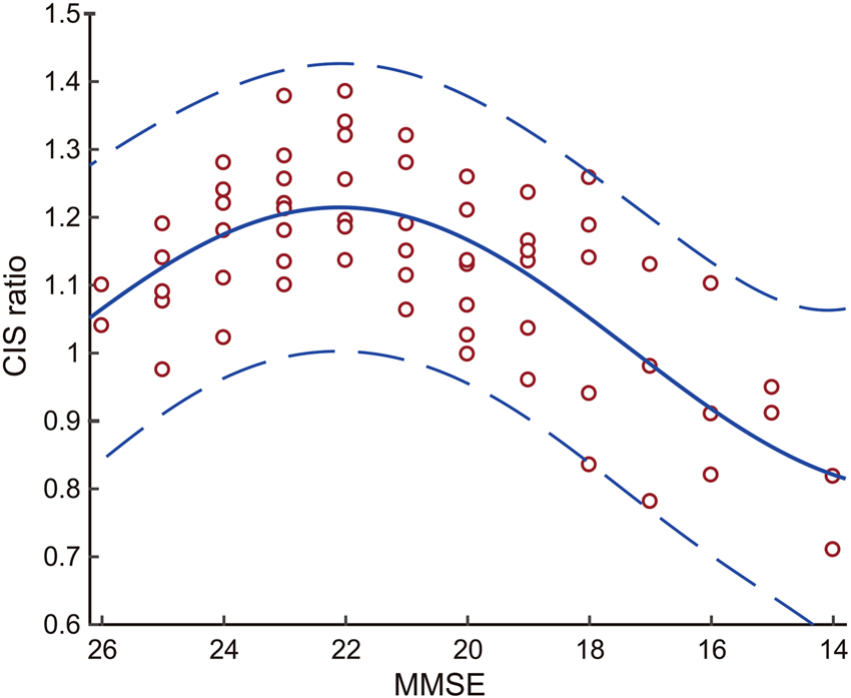
Inverted U-shaped relationship between MMSE scores and CIS ratios at 1st and 2nd SPECT evaluations. The regression curve estimated by Curve Fitting Toolbox on MATLAB showed an inverted U-shaped relationship between the CIS ratio and MMSE. The CIS ratio peaked at an MMSE score of 22.1 on the estimated curve (solid line) [f(x) = 1.006 + 0.1133cos(0.3293x) + 0.1748sin(0.3293x): *R* ^2^ = 0.5333, DFE = 58, adjusted *R* ^2^ = 0.5091]. The upper and lower broken lines represent 95% prediction bounds.

**Figure 6:**
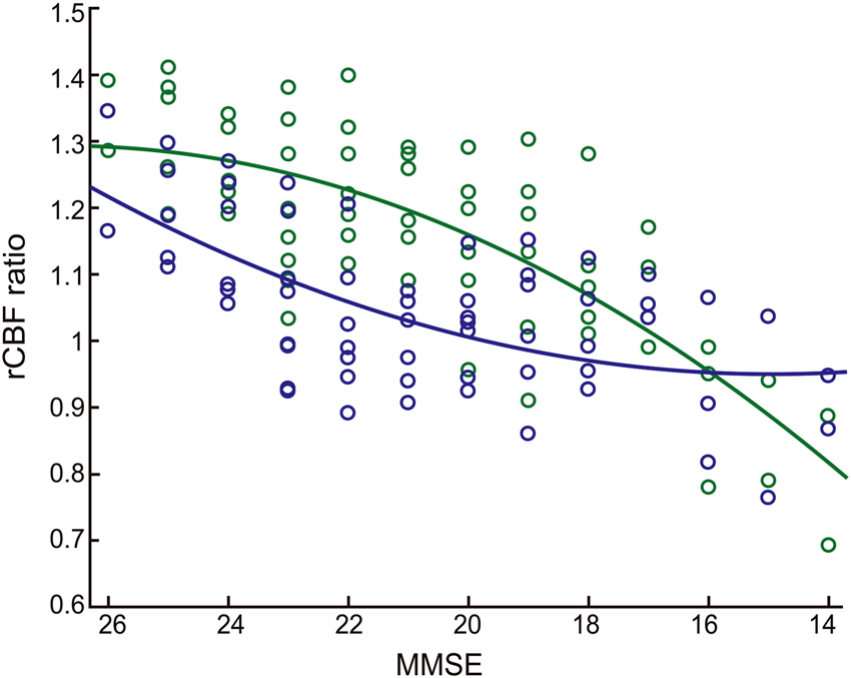
MMSE scores and rCBFratio in PpC and PCC. The estimated curve for PCC (*green*) is [f(x) = −0.7978 − 2.026 × 10^7^(1 − cos(−1.696 × 10^−5^x)) − 9205 sin(−1.696 × 10^−5^x): *R* ^2^ = 0.5963, DFE = 58, adjusted *R* ^2^ = 0.5754]. The curve for PpC (*blue*) is [f(x) = 1.4323 + 2.867 × 10^6^(1 − cos(3.897 × 10^−5^x)) − 1670sin(3.897 × 10^−5^x): *R* ^2^ = 0.3920, DFE = 58, adjusted *R* ^2^ = 0.3605]. Curves for PpC and PCC start to decrease early and later, respectively, during DLB progression. Note that PCC curve is convex upward and PpC curve is convex downward.

The regression curve estimated by Curve Fitting Toolbox on MATLAB showed an inverted U-shaped relationship between the CIS ratio and MMSE (Fig. 5). The CIS ratio peaked at an MMSE score of 22.1 on the estimated curve [f(x) = 1.006 + 0.1133cos (0.3293x) + 0.1748sin (0.3293x): *R* ^2^ = 0.5333, DFE = 58, adjusted *R* ^2^ = 0.5091]. Broken lines in Fig. 5 denote 95% prediction interval. The estimated curve of rCBF ratio differed between the PpC and PCC (Fig. 6). That is, the rCBF ratio in PpC began to decrease at the earlier stages of DLB and that in PCC decreased later.

## Discussion

The present study found that the CIS ratio decreased as mild DLB progressed, but increased in patients with prodromal DLB during two-year follow-up. Our hypothesis that CIS disappears as DLB progresses was demonstrated. It would be attributed to AD-type NFT pathology increasing over time. MTL shrinkage over time would support the view. The relationship between the CIS ratio and the MMSE score, which decreased in all patients with DLB during follow-up, was inverted U-shaped. The CIS ratio peaked at an MMSE score of 22.1 on the regression curve and diminished as MMSE decreased. Decreases in MMSE scores and MTL volumes were more obvious among mild DLB patients with lower initial CIS ratios than higher initial CIS ratios. Thus, the CIS changed over time and was most obvious in the vicinity of an MMSE score of 22. The finding of a lower CIS ratio in mild DLB predicts the progression of cognitive decline.

The inverted U-shaped relationship between CIS ratio and MMSE scores might have caused the failure of linear analysis of previous studies. The results of our previous linear correlation analysis showed that the CIS ratio in mild DLB did not correlate with MMSE scores^18^. The CIS ratio was not reported to correlate with the Mattis Dementia Rating Scale, either^19^. The inverted U-shape was constructed with CIS ratios that increased or decreased as MMSE decreased during two-year follow-up (Figs 2, 3 and 4). The temporal changes in the CIS ratio with disease progression should be taken into consideration when basing a differential diagnosis on CIS. The CIS seems to be able to discriminate DLB from AD more effectively than only occipital hypometabolism or hypoperfusion^16,17^. Certainly positive CIS may predict probable DLB. However, our study indicates that advanced or prodromal DLB may not show obvious CIS. Before the CIS can be used as a biomarker to distinguish DLB from AD, the longitudinal behavior of the CIS in DLB should be defined.

The formation of the inverted U-shape of CIS ratio, which derived from dividing the rCBF ratio in the PCC by that in the PpC, can be explained by difference in temporal change between the rCBF ratio in the PCC and that in the PpC. Figure 6 shows different regression curves for the rCBF ratio in the PpC and PCC. Both rCBF ratio in PCC and PpC were relatively high at the prodromal stage. The rCBF ratio in the PpC began to decrease in the PpC earlier than that in the PCC, and both gradually changed as MMSE scores decreased, which rendered the CIS more obvious in mild DLB. When MMSE decreased to fewer than 20, the regression curves of rCBF ratio in PCC and PpC again became closer. Thus, the relationship between the CIS ratios and MMSE scores became an inverted U-shape. Furthermore, the pathological features of DLB is constructed with a mixture of Lewy body and AD-type NFT pathology and the pathological basis of hypoperfusion in the PCC and PpC were AD-type NFT and Lewy body pathologies, respectively^23,24^. Therefore, the difference in regression rCBF ratio curves between PpC and PCC was attributed to differences in how Lewy body- and AD-pathologies progress. The variability of CIS is considered to be based on these pathological differences.

Concomitant AD-type NFT pathology will account for our finding indicating more rapid cognitive decline in a lower, than a higher initial CIS ratio in mild DLB. As the CIS has been reported to be influenced AD pathology^18,19^, we grouped the patients with mild DLB according to whether progression differed based on higher or lower initial CIS ratios that reflect lower and higher degrees of AD pathology, respectively. As most patients with DLB also have various degrees of concomitant AD pathology, pure DLB is relatively uncommon^25^. Any patients with pure DLB in the present study were likely to have higher CIS ratios. Previous studies have shown a worse prognosis of DLB with AD pathology than pure DLB, e.g. more rapid cognitive decline^26,27^, worse response to acetylcholinesterase inhibitor^28^ and lower survival rates^29,30^. The reported annual reduction in MMSE scores of patients with AD is 3.7 points^31^. Having a higher and a lower initial CIS ratio was associated with annual reductions of 1.5 and 2.5 points, respectively. Thus, cognitive decline in patients with DLB who had a lower initial CIS ratio more similarly progressed to patients with AD. The finding was consistent with previous report that presence of abundant AD-type NFT pathology in addition to Lewy body pathology made the clinical profile more resemble AD rather than DLB^32^. In line with above discussion, the finding of a lower CIS ratio in mild DLB predicts the progression of cognitive decline.

CIS is not specific to DLB, although it is commonly observed in mild DLB cases. Posterior cortical atrophy also shows CIS^33^ in spite of abundant AD-type NFT pathology. The assertion that higher Braak NFT stage leads to smaller CIS ratio^19^ is correct in DLB cases, but is not applied to posterior cortical atrophy. The NFT distribution of posterior cortical atrophy often shows relative sparing of the hippocampus. We have previously demonstrated that the CIS in DLB was associated with MTL atrophy^18^. Therefore, CIS is considered to reflect sparing of posterior limbic circuitry and it is worth keeping in mind that CIS cannot be a reliable sign for Lewy body pathology.

We have successfully delineated CIS with ^123^I-IMP SPECT. The appearance of the CIS on FDG-PET was initially described as being specific to DLB and it has been considered as a useful hallmark, especially of pure DLB^16,18,19^. The CIS ratio on ^99m^Tc-ethyl cysteinate dimer (ECD) perfusion SPECT can also discriminate DLB from AD^17^, although spatial resolution of PET is superior to SPECT. This seems natural, because both the glucose metabolism and CBF reflect brain function^34^. Furthermore, ^123^I-IMP has a higher first-pass extraction than other brain perfusion SPECT tracers such as HMPAO and ECD^35,36^ and it can show contrast particularly at the high range of CBF such as PCC and PpC where the CIS produced. Therefore, ^123^I-IMP seems suitable for detecting small changes in CIS, although the image quality afforded by ^99m^Tc-HMPAO or ^99m^Tc-ECD is superior because higher doses of ^99m^Tc can be administered.

This study has several limitations. Firstly, the diagnosis of DLB was not confirmed at autopsy. However, all patients with DLB in this study had probable DLB based on strict international workshop DLB criteria^37^ and showed decreased DAT density on DAT-SPECT. As for patients with prodromal DLB, all of them developed mild DLB and were also confirmed decreased DAT density during two-year follow up. Secondly, this study proceeded at a single clinic, and thus the sample of patients was not large. Further study with larger size would be required.

## Conclusion

The CIS on IMP-SPECT changed over time and was the most obvious in the vicinity of an MMSE score of 22 in patients with DLB. Having its peak seemed a distinctive feature of CIS, as most biomarkers became more obvious as disease progressed. A lower CIS ratio in mild DLB was associated with a worse prognosis for cognitive decline, presumably due to concomitant AD pathology that increases over time.

## Methods

### Patients

The present study included 24 patients with mild DLB were selected at Fukujuji Hospital, Tokyo between 2011 and 2014 by following criteria: 1) over 65 of age; 2) diagnosed with probable DLB according to the DLB consensus criteria^1^; 3) the diagnosis of mild DLB was based on the McKeith criterion for probable DLB, namely, at least two core symptoms^1^; 4) having 1 of clinical dementia rating scale score; 5) abnormal finding on DAT-SPECT. Seven other patients who were diagnosed with prodromal DLB^38^ were also enrolled at Fukujuji Hospital, Tokyo between 2011 and 2014 by following criteria: 1) over 65 of age; 2) prodromal DLB was defined as mild cognitive impairment according to the Petersen criteria^39^, with preserved independence assessed using the Instrumental Activities of Daily Living and meeting the McKeith criteria for probable DLB except for the presence of dementia^1^; 3) having 0.5 of clinical dementia rating scale score; 4) developed mild DLB during two-year follow-up; 5) abnormal finding on DAT-SPECT during two-year follow-up. We also included 22 persons with normal cognition. Cognitive function was assessed using the MMSE. The patients with DLB were assessed twice by ^123^I-IMP-SPECT and MRI at an interval of two years. The persons with normal cognition underwent ^123^I-IMP once. Cerebral vascular disease and other neurodegenerative diseases were ruled out by MRI. This study followed the clinical study guidelines of Fukujuji hospital, which conformed to the Helsinki Declaration. All procedures were approved by the hospital Ethical Review Board. We provided patients and their families with detailed information, and written informed consent was obtained from all participants.

### Brain perfusion SPECT imaging

Patients resting with their closed eyes and their ears unplugged were assessed twice at an interval of two years using ^123^I-IMP and an E-CAM gamma camera (Toshiba Medical Systems Corporation, Otawara, Japan) with fan beam collimators. Starting from 15 min after an intravenous infusion of 167MBq of ^123^I-IMP, SPECT images were acquired in a 128 × 128 matrix with a slice thickness of 1.95 mm (1 pixel) over a period of 30–40 min. The images were reconstructed by filtered back projection using a Butterworth filter, attenuation was corrected using the Chang method (attenuation coefficient = 0.1 cm^−1^) and scatter was corrected using a triple energy window as described^40^.

### VSRAD analysis

All patients were assessed with 1.5 T MRI scanner (Magnetom Aera; Siemens, Erlangen, Germany). Three-dimensional volumetric acquisition of a T1-weighted gradient echo sequence was used to produce a gapless series of thin sagittal sections using a magnetization preparation rapid acquisition (repetition time 1700 ms, echo time 4.0 ms, flip angle 15°, acquisition matrix 256 × 256, 1.3 mm slice thickness). The VSRAD analysis was performed as described by Matsuda *et al*.^41^ and evaluates the atrophy of MTL that involved the entire region of the entorhinal cortex, hippocampus and amygdala. The degree of atrophy is expressed as VSRAD score (Z score) relative to a normal database bundled with VSRAD advance software (Eisai, Tokyo, Japan) that comprises 80 healthy volunteers (37 men and 43 women; age, 54–86 year).

### Image analysis

Three-dimensional stereotactic surface projections created using Neurological Statistical Image Analysis Software developed by Minoshima *et al*. were applied to the ^123^I-IMP SPECT images to generate 3D CBF images and Z-score maps^42^. We applied region of interest (ROI) analysis to measure rCBF in the PCC, precuneus and cuneus using stereotaxic extraction estimation version 2.1 software (Nihon Medi-Physics Co. Ltd., Tokyo, Japan)^43^. The mean rCBF in each segment was automatically measured after segmentation based on anatomical classification of the standard brain. We selected segments of the PCC (Brodmann 23 and 31), precuneus and cuneus (Supplementary Fig. 1). The mean value in the bilateral PCC ROI was divided by the mean value in the bilateral PpC ROI to derive CIS ratios from IMP-SPECT images. The rCBF in the PCC and PpC was divided by the mean brain CBF to derive rCBF ratio. The ^123^I-IMP SPECT images were spatially normalized using SPM12 (Wellcome Department of Cognitive Neurology, London, UK) and rCBF change between 1st and 2nd SPECT images was assessed with paired *t*-test.

### Statistical analysis

Temporal changes in CIS ratio, MMSE scores and MTL volumes during two-year follow-up in each group were evaluated by paired *t*-test. Changes in MMSE scores and MTL volumes were also compared between patients with mild DLB and higher and lower initial CIS ratios using a repeated measures ANCOVA. Moreover, data from all patients were assessed using multiple regression analysis to predict change in CIS ratio based on selected explanatory variables. The candidates for the variables were age, gender, years of education, 1st MMSE at 1st SPECT, 1st MTL atrophy and DAT density. We applied forward-backward stepwise procedure for variable selection to find a model with maximum adjusted *R* ^2^. All statistical tests were two-sided, and data were statistically analyzed using SPSS (SPSS Inc., Chicago, IL, USA) except curve fitting. The curve estimation was performed with Curve Fitting Toolbox on MATLAB (MathWorks Inc., MA, USA).

## Electronic supplementary material

Supplementary Figure 1
