# Supplementary material for: Cingulate island sign temporally changes in dementia with Lewy bodies
Source: Sci Rep. 2017 Nov 7;7:14745. doi: 10.1038/s41598-017-15263-2 (PMC5677123; doi:10.1038/s41598-017-15263-2)
Supplement: Supplementary file 1 — Supplementary Figure 1 [file 41598_2017_15263_MOESM1_ESM.pdf]

Cingulate island sign temporally changes in dementia with Lewy bodies

Tomomichi Iizuka,[1] Rui Iizuka[2] and Masashi Kameyama[3,4]

[1]Center for Dementia, Fukujuji Hospital, Japan Anti-Tuberculosis Association, 24-1-3, Matsuyama, Kiyose-City, Tokyo, 204-8522, Japan.

[2]Department of Biology, Waseda University, 1-104 Totsukamachi, Shinjuku-ku, Tokyo, 169-8050, Japan.

[3]Division of Nuclear Medicine, Department of Radiology, School of Medicine, Keio University, 35 Shinanomachi, Shinjuku-ku, Tokyo 160-8582, Japan

[4]Department of Radiology, Tokyo Metropolitan Geriatric Hospital, 35-2 Sakaecho, Itabashi-ku, Tokyo, 173-0015, Japan

Supplementary Figure Legends

Supplementary Figure 1. ROIs for rCBF evaluation

The ROIs for PCC, Precuneus and Cuneus are shown. The PCC ROI consists of Brodmann area 23 and 31.

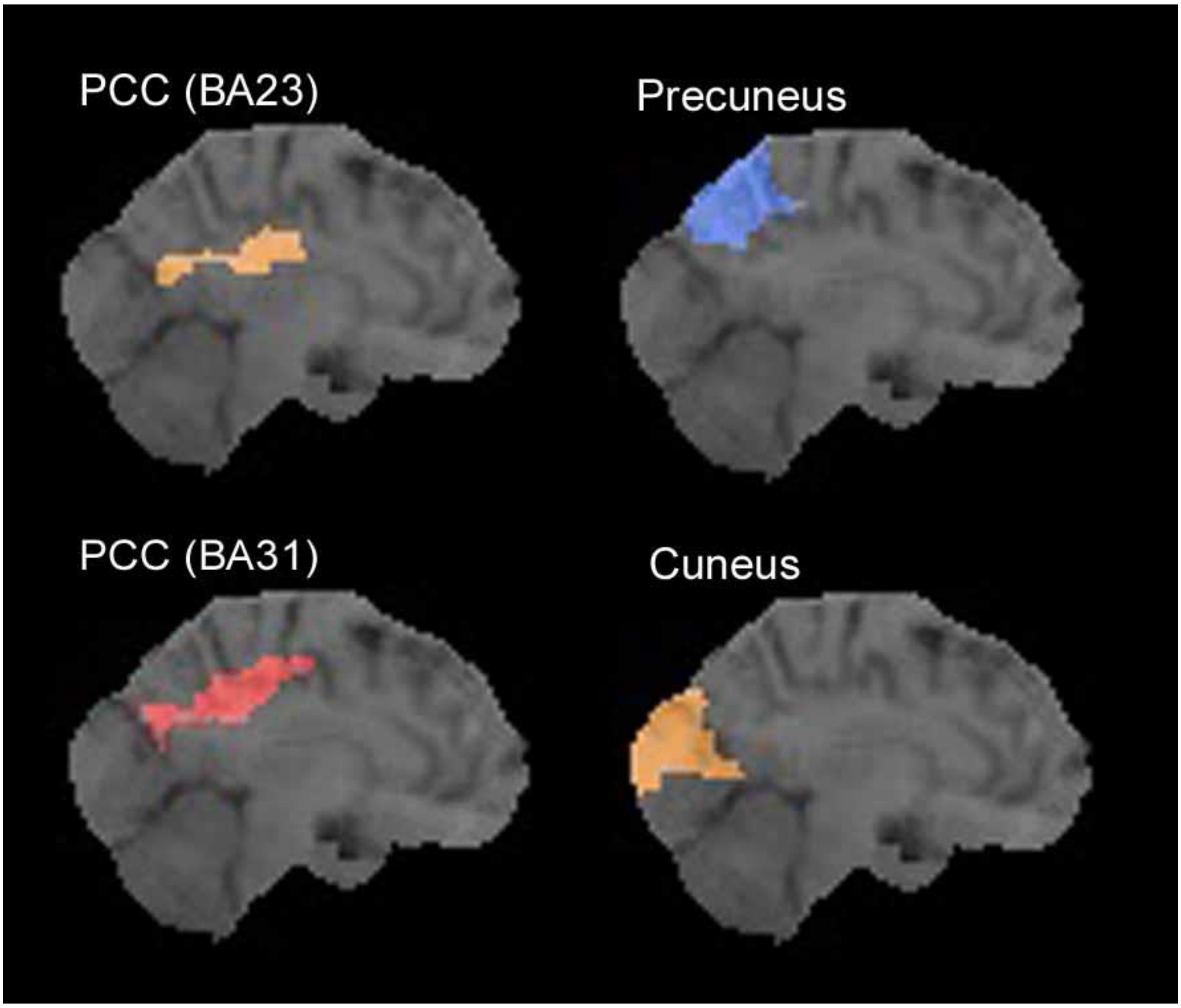
PCC (BA23)
Precuneus
PCC (BA31)
Cuneus
